# Gliding Motility and Expression of Motility-Related Genes in Spreading and Non-spreading Colonies of *Flavobacterium columnare*

**DOI:** 10.3389/fmicb.2018.00525

**Published:** 2018-03-26

**Authors:** Reetta Penttinen, Ville Hoikkala, Lotta-Riina Sundberg

**Affiliations:** Centre of Excellence in Biological Interactions, Department of Biological and Environmental Science and Nanoscience Center, University of Jyväskylä, Jyväskylä, Finland

**Keywords:** colony type, *Flavobacterium columnare*, gene expression, gliding motility, nutrients, RT-qPCR, T9SS, type IX secretion system

## Abstract

Gliding motility facilitates the movement of bacteria along surfaces in many Bacteroidetes species and results in spreading colonies. The adhesins required for the gliding are secreted through a gliding motility-associated protein secretion system, known as the type IX secretion system (T9SS). The fish pathogen *Flavobacterium columnare* produces spreading (rhizoid [Rz], soft [S]) and non-spreading (rough [R]) colony types, of which only the spreading Rz type is virulent. In this study, we explored the spreading behavior of these colony types by microscopic imaging and measured the expression of genes associated with gliding motility and T9SS (*gldG, gldH, gldL, sprA, sprB, sprE, sprF, sprT*, and *porV)* under high and low resource levels by using RT-qPCR (reverse transcription quantitative PCR). The spreading colony types responded to the low resource level with increased colony size. The non-spreading colony type, as well as the cells growing under high nutrient level expressed only moderate cell movements. Yet, a low nutrient level provoked more active gliding motility in individual cells and increased spreading by cooperative gliding. The gene expression survey demonstrated an increased expression level of *sprA* (a core component of T9SS) and *sprF* (needed for adhesin secretion) under low nutrient conditions. Surprisingly, the expression of gliding motility genes was not consistently associated with more active spreading behavior. Furthermore, no genetic differences were found between spreading and non-spreading colony types in the studied genes associated with gliding motility. Our study demonstrates that environmental nutrient level is an important regulator of both gliding motility and the expression of some of the associated genes. These results may help to understand the connections between nutrient concentration, gliding motility, and virulence of *F. columnare*.

## Introduction

Gliding motility is a process of bacterial movement on surfaces exploited by several bacterial species in the phylum Bacteroidetes (McBride and Zhu, 2013). Instead of involving flagellae or pili, gliding motility is enabled by complex machinery that has been studied more closely in *Flavobacterium johnsoniae* (for a review of *Flavobacterium* gliding motility, see McBride and Nakane, 2015), a model system for gliding motility. Number of studies of flavobacterial gliding motility have led to the identification of several genes involved in motility, including *gldA, gldB, gldD, gldF, gldG, gldH, gldI, gldJ, gldK, gldL, gldM, gldN, sprA, sprB, sprE*, and *sprT* (Agarwal et al., 1997; Hunnicutt and McBride, 2000, 2001; Hunnicutt et al., 2002; McBride et al., 2003; McBride and Braun, 2004; Braun and McBride, 2005; Nelson et al., 2007, 2008; Sato et al., 2010; Rhodes et al., 2010, 2011b; Kharade and McBride, 2015). Furthermore, a subset of these genes, *gldK, gldL, gldM, gldN, sprA, sprE*, and *sprT*, has been found to compose a protein translocation system, designated the type IX secretion system (T9SS) (Sato et al., 2010; McBride and Zhu, 2013). T9SS-related genes are restricted to Bacteroidetes, with no prevalent similarity between the previously described secretion systems (McBride and Zhu, 2013). T9SS has an important role in the secretion of gliding motility adhesins, being required both for surface contact and for secretion of virulence factors (Sato et al., 2010; Shoji et al., 2011). For example, the periodontal pathogen *Porphyromonas gingivalis* uses T9SS for secretion of its major virulence factors, which are gingipains and hemagglutinins (Sato et al., 2010; Shoji et al., 2011).

The exact role of each component in the gliding motility machinery of *F. johnsoniae* is not yet fully understood. GldB, GldD, GldH, GldI, and GldJ are lipoproteins needed for gliding, but their exact functions are not known (Hunnicutt and McBride, 2000, 2001; McBride et al., 2003; McBride and Braun, 2004; Braun and McBride, 2005). GldA, GldF, and GldG form an ABC transporter but its role in gliding is still largely unknown (Agarwal et al., 1997; Hunnicutt et al., 2002). *sprB* is a motility adhesin needed for gliding, and it is secreted via the T9SS (Sato et al., 2010). The SprB encoding gene is located in operon *sprCDBF*, where it is transcribed together with *sprC* and *sprD*, genes coding for proteins that support SprB function, and with *sprF*, which is needed for successful secretion of SprB (Rhodes et al., 2011a). In addition, a recently identified gene, *porV*, is needed for secretion of chitinase and adhesin RemA in *F. johnsoniae* (Kharade and McBride, 2015). The mechanisms that control the assembly and activity of gliding motility machinery and T9SS are not known. In *P. gingivalis*, a two-component regulative system consisting of PorX and PorY regulates the expression of a subset of T9SS genes (Sato et al., 2010).

*Flavobacterium columnare* is a fish pathogen belonging to the phylum Bacteroidetes. *F. columnare* carries the majority of the orthologous genes (Tekedar et al., 2012) involved in flavobacterial gliding motility and T9SS, which are used for virulence factor secretion and formation of spreading colonies (Sato et al., 2010; McBride and Nakane, 2015). *F. columnare* can form different colony morphotypes, including the spreading rhizoid (Rz) and soft (S) colony types as well as the non-spreading rough (R) type (Kunttu et al., 2009; Laanto et al., 2012). Spreading colony morphology has been suggested to be essential for *F. columnare* virulence (Kunttu et al., 2009; Laanto et al., 2012), and indeed, only the spreading Rz type is virulent in the fish host (Kunttu et al., 2009; Laanto et al., 2012, 2014). Furthermore, changes in nutrient concentration in agar culture changes spreading behavior of *F. columnare* colonies, especially in the virulent Rz type (Laanto et al., 2012). Nutrient availability also has a significant impact on virulence in *F. columnare* as a high nutrient level induces higher virulence in the bacteria (Penttinen et al., 2016; Kinnula et al., 2017). The functionality of gliding motility and T9SS in different *F. columnare* morphotypes is not known, although *gldL*, *gldM*, *gldN*, and *gldH* have been suggested as putative virulence-associated factors in *F. columnare* (Dumpala et al., 2010; Klesius et al., 2010). Indeed, a recent paper by Li et al. (2017) shows decreased virulence in a secretion-deficient T9SS mutant. Furthermore, a transcriptome-wide study of *F. columnare* strain ATCC 49512 demonstrated that genes associated with gliding motility and spreading are located in actively transcribed operons (Tekedar et al., 2017). Yet, there is a significant gap in the current understanding of the genetic factors underlying the virulent and non-virulent colony morphologies. In addition, how the environmental conditions regulate the gliding motility and expression of the T9SS in *F. columnare* has remained poorly understood. These issues have to be clarified in order to understand pathogenesis of *F. columnare*.

Here, we explored gliding motility in *F. columnare* spreading (Rz, S) and non-spreading (R) morphotypes under conditions that were expected to induce (low-nutrient) or reduce (high-nutrient) spreading behavior. Gliding motility and individual cell movements were seen to be more active under low-nutrient conditions. We also performed a RT-qPCR assay in order to measure the gene expression of T9SS or gliding motility -associated genes *gldG, gldH, gldL, sprA, sprB, sprE, sprF, sprT*, and *porV*. Of these genes, *gldL, porV, sprA, sprE*, and *sprT* are associated with the T9SS. Increased gene expression in response to low nutrient availability was detected in *sprA* and *sprF.* However, the spreading and non-spreading colony types had different expression profiles under different resource levels, which could be an indication of divergent metabolic programs.

## Results

### Nutrient Availability Regulates Colony Spreading in Rz and S Morphotypes

**FIGURE 1:**
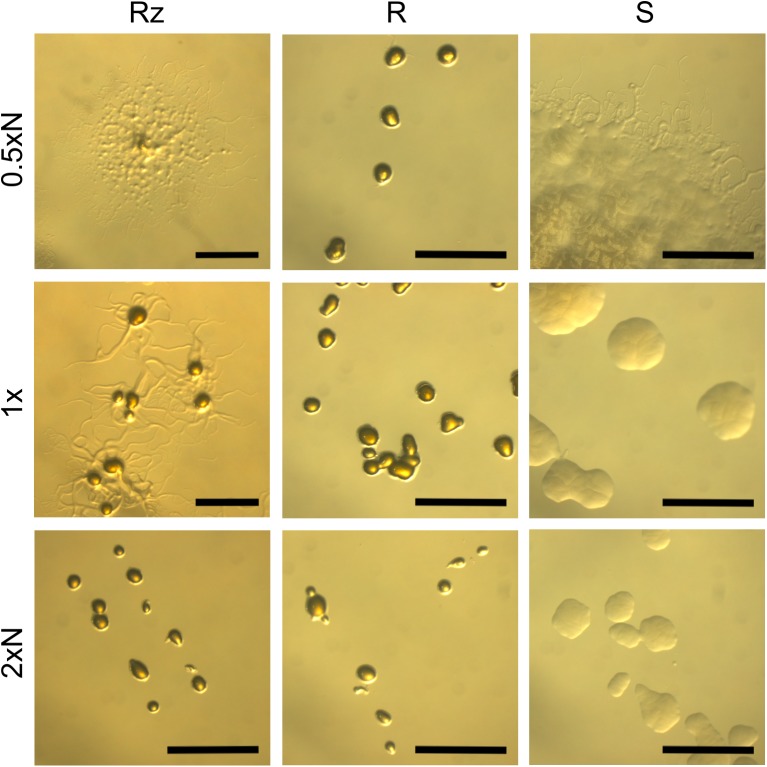
*F. columnare* B067 colony types Rz, R, and S growing on 0.5xN, 1x, and 2xN Shieh agar plates. N refers to peptone and yeast extract concentrations which were either halved (0.5xN), normal (1x), or doubled (2xN). See **Figure 2A** for the mean colony sizes. Scale bars 1 mm.

**FIGURE 2:**
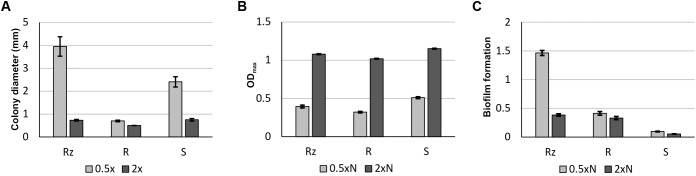
Growth characteristics of *F. columnare* B067 colony types Rz, R, and S in different nutrient levels. Mean colony size in millimeters (± SE) of colonies growing on 0.5x and 2x Shieh agar plates **(A)**. Maximum optical density (at 600 nm) (± SE) reached during a 65-h-liquid cultivation in 0.5xN and 2xN Shieh **(B)**. Biofilm formation (at 595 nm) (± SE) after 44 h in 0.5xN and 2xN Shieh **(C)**.

The morphology of bacterial colonies was assayed on 0.5xN, 1x and 2xN Shieh plates, as these nutrient levels have previously been shown to be useful for exemplifying gliding motility in *F. columnare* (Laanto et al., 2012). The bacteria originating from the same liquid culture were spread on agar plates and grown for 2 days after which the colony morphology was imaged under a light microscope (**Figure 1**). Rz colonies grown on a 0.5xN Shieh plate were spreading with increased mean colony size (3.95 mm, SE ±0.42) and the production of root-like protrusions typical for Rz morphology (**Figures 1**, **2A**). Rz colonies grown on 2xN Shieh plates had a smaller mean colony size (0.73 mm, SE ±0.04), and root-like structures, if seen, were only moderate (**Figures 1**, **2A**). Type S responded to changing nutrient availability comparably to Rz (mean colony size 2.4 mm, SE ±0.23 in 0.5xN and 0.75 mm, SE ±0.06 in 2xN). However, when grown at lower nutrient conditions (0.5xN Shieh), root-like structures were also observed in the S type. These colonies were, nevertheless, distinguishable from Rz colonies by their non-adherent, opaque, and moist appearance (**Figure 1**). R type did not remarkably alter the colony size in response to changing nutrient availability (mean colony size 0.7 mm, SE ±0.03 in 0.5xN and 0.5 mm, SE ±0.00 in 2xN) and root-like structures were only occasionally seen under low nutrient conditions (**Figures 1**, **2A**).

### Growth and Biofilm Formation in Varying Nutrient Concentrations

The viability of Rz, R, and S colony types in 0.5xN and 2xN Shieh media was measured as the maximum optical density (OD*_max_*) reached during a 65-h cultivation period (**Figure 2B**). All colony types reached the highest OD*_max_* at higher nutrient level (2xN Shieh). The biofilm forming ability was remarkably higher under low nutrient conditions (0.5xN Shieh) in the Rz type compared to 2xN Shieh as well as to the R and S types, which were weaker biofilm producers at both nutrient levels (**Figure 2C**).

### Imaging of Bacterial Movements

The movements of individual Rz cells grown on 0.5xN and 2xN Shieh agar plates were recorded with a confocal microscope. The movements of Rz cells were comparable with the previously described gliding motility of *F. johnsoniae*; the cells glided over the surface in a straightforward manner, occasionally attaching to the surface with one end of the cell, rotating, and changing the moving direction (Supplementary Videos 1, 2). The gliding speed was slower than that seen in *F. johnsoniae*, which was used as a reference (data not shown).

**FIGURE 3:**
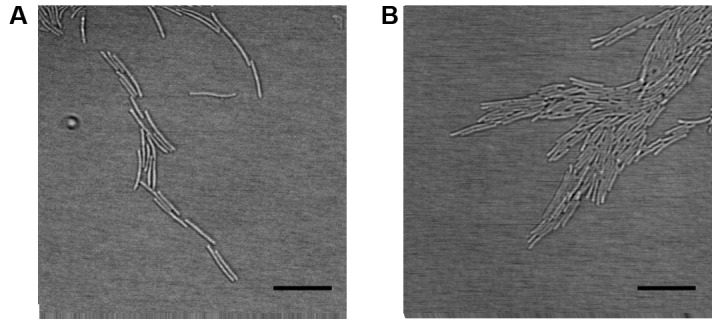
Organization of *F. columnare* B067 cells as microrhizoids on the edges of biofilm. Rz colony type grown under 1x Shieh agar layer **(A)** and S type under 0.5xN Shieh agar **(B).** Scale bars 10 μm.

The movements of individual bacteria growing as a part of forming biofilm were recorded between the agar layer and microslide chamber bottom. The colony types Rz and S formed a monolayer on the edges of spreading bacterial biofilm, where the cells were organized side by side and glided along the adjacent cells (**Figure 3** and Supplementary Videos 3, 4, 7, 8). The cells formed branching rhizoid-like structures a few cells wide (here referred to as microrhizoids), which involved both motile and non-motile cells. In both Rz and S types, more active gliding motility was seen under low nutrient concentrations (0.5xN). Colony type R expressed only occasional movements regardless of the nutrient level, and cellular organization as a spreading biofilm was not observed (Supplementary Videos 5, 6). In order to visualize *F. columnare* colony formation on a longer timescale, the growth of the Rz colony type on 1x Shieh was recorded over the course of 8 h (Supplementary Video 9). In the front of the biofilm, the bacteria were characteristically organized in microrhizoids, which moved cooperatively toward the spreading direction of the biofilm and seemed also to serve as routes along which other cells were able to glide further and support biofilm expansion.

### Sequence Analysis and Expression of Gliding Motility-Associated Genes

**FIGURE 4:**
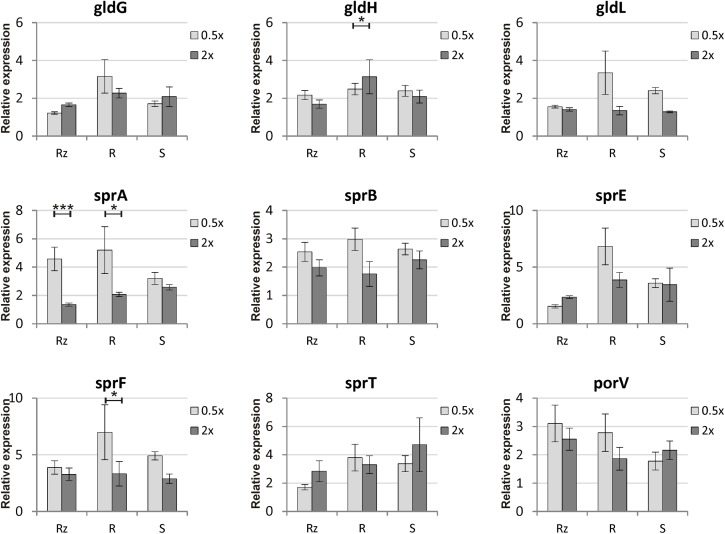
Relative gene expressions of gliding motility and T9SS genes (± SE) in *F. columnare* B067 colony morphologies Rz, R and S cultivated on 0.5x and 2x Shieh agar plates. The concentration of all Shieh components was either halved (0.5x) or doubled (2x) compared to the normal Shieh medium. For detailed statistical analysis of the gene expression results, see **Tables 1**, **2**. Asterisks indicate the statistical significance (*p*-values) between treatments: ^∗^*p* < 0.05; ^∗∗^*p* < 0.01; ^∗∗∗^*p* < 0.001.

Genes putatively involved in gliding motility were sequenced from Rz, R, and S types of strain *F. columnare* B067. No genetic differences were detected between the colony types in the gene sequences of operons *gldFG* and *gldKLMN*, genes *gldH*, *sprA, sprC, sprD, sprE, sprF, sprT, porV, porX*, and *porY*, or in the predicted regulative regions upstream from the genes *gldH, sprA, sprE*, and *porV* or operons *gldFG*, *gldKLMN*, and *sprCDBF*. Expression of genes *gldG, gldH, gldL, sprA, sprB, sprE, sprF, sprT*, and *porV*, which are putatively involved in *F. columnare* gliding motility, was measured in B067 Rz, R, and S colony types that had been grown on 0.5x and 2x Shieh agar plates. Of these genes, *gldL, sprA, sprE, sprT*, and *porV* are associated with the T9SS. Gene expression results were normalized with reference genes *gapdh* and *glyA*, which are stably expressed in the current dataset (the M value was 0.5834 for both genes, with a variance coefficient of 0.2114 for *gapdh* and 0.2007 for *glyA*). Relative expressions are presented in **Figure 4** (for statistics, see **Tables 1**, **2**). Significant differences between colony types were observed in the expression of genes *gldG, gldH, gldL*, and *sprE* (**Table 1** and **Figure 4**; for pairwise comparisons, see **Table 2**). Nutrient levels had a significant effect on *gldL, sprA*, *sprB*, and *sprF* expression. The pairwise comparisons revealed that *sprA* was expressed at significantly higher levels in low-nutrient conditions in Rz and R types, and the same pattern was detected in *sprF* expression in the R type (**Table 2** and **Figure 4**). However, significant interaction between the colony type and nutrient was detected in *gldH*, *gldL, sprA*, and *sprE* (**Table 1**), indicating that gene expression of colony types may differ between nutrient conditions. Indeed, direct associations between spreading behavior and gliding motility gene expression were challenging to form as different colony types seemed to respond differently to the nutrient level with motility gene expression. Even though a significant effect of colony type was not observed in either *sprT* or *porV*, a significant difference between Rz and R was observed in *sprT* and between Rz and S in *porV* expression (**Table 2**).

**Table 1:** Statistical analysis of gliding motility and T9SS gene expression of *F. columnare* B067 Rz, R and S colony types grown on 0.5x and 2x Shieh agar plates.

Gene	Colony type			Nutrient			Colony type^∗^ nutrient interaction		
			
	*F*	*df*	*p*	*F*	*df*	*p*	*F*	*df*	*p*
*sprB*	0.225	2	0.800	6.583	1	0.016	0.185	2	0.832
*gldG*	10.148	2	<0.001	0.523	1	0.476	1.289	2	0.291
*gldH*	6.093	2	0.006	0.229	1	0.636	3.918	2	0.032
*gldL*	7.118	2	0.003	19.605	1	<0.001	5.912	2	0.007
*sprT*	3.063	2	0.063	0.469	1	0.499	0.519	2	0.601
*porV*	2.268	2	0.122	0.654	1	0.426	0.493	2	0.616
*sprA*	0.807	2	0.456	21.363	1	<0.001	4.433	2	0.021
*sprE*	11.647	2	<0.001	0.448	1	0.509	3.382	2	0.048
*sprF*	1.681	2	0.204	7.312	1	0.012	1.072	2	0.356


**Table 2:** Pairwise comparisons of gliding motility gene expression between *F. columnare* B067 Rz, R and S colony types and between growth conditions 0.5x and 2x Shieh agar within a colony type.

Gene	Pairwise comparisons of colony types			Nutrient 0.5x vs. 2x		
		
	*Rz vs. R*	*Rz vs. S*	*R vs. S*	*Rz*	*R*	*S*
*sprB*	NS	NS	NS	NS	NS	NS
*gldG*	<0.001	NS	0.004	NS	NS	NS
*gldH*	0.002	NS	0.009	NS	0.028	NS
*gldL*	0.0108	0.0087	NS	NP	NP	NP
*sprT*	0.030	NS	NS	NS	NS	NS
*porV*	NS	0.043	NS	NS	NS	NS
*sprA*	NS	NS	NS	<0.001	0.019	NS
*sprE*	<0.001	0.025	0.007	NS	NS	NS
*sprF*	NS	NS	NS	NS	0.041	NS


*NS: statistically not significant result when confidence level of 0.95 was used; NP: not performed.*

### Proteolytic Activity and Extracellular Secretion in Different Nutrient Concentrations

Colony types Rz, R, and S were cultivated on 0.5xN and 2xN Shieh plates containing 1.5% skim milk. Proteolytic activity was observed in each colony variant, seen as the formation of a clear degradation zone peripheral to the bacterial growth, but no differences between the colony types were observed, and the nutrient concentration did not affect the proteolytic activity (Supplementary Figure S1). The effect of colony type and nutrient availability on the contents of extracellularly secreted products (ECPs) was analyzed further. Generally, Rz, R, and S grown in 0.5xN and 2xN liquid Shieh cultures shared a common overall ECP profile, with some moderate changes in individual protein bands between colony types (Supplementary Figure S2). However, a strong protein band, approximately 13 kD in molecular weight, was detected in Rz type grown in both 0.5xN and 2xN Shieh media. The corresponding band was absent or barely detectable in R or S types.

## Discussion

The ability to move toward nutrient sources and the host is essential for the survival of bacteria, and bacterial virulence has been shown to be associated with motility in several bacterial species (e.g., Josenhans and Suerbaum, 2002; McGee et al., 2002; Haiko and Westerlund-Wikstrom, 2013). Comparative genomic analysis has revealed that several members of Bacteroidetes employ a unique gliding motility machinery and a motility-related secretion system T9SS (McBride and Zhu, 2013). Cells possessing a functional gliding motility system characteristically form spreading colonies (McBride and Nakane, 2015). *F. columnare* and *F. psychrophilum* are pathogenic in fish, affecting freshwater aquaculture on a global scale (Wagner et al., 2002; Declercq et al., 2013; Nilsen et al., 2014). It has been suggested that T9SS may have a central role in the pathogenesis of these species (Laanto et al., 2012; Castillo et al., 2015). This was confirmed recently with T9SS-mutant *F. columnare*, which was demonstrated to have a decreased level of virulence (Li et al., 2017).

Here, we imaged the gliding motility of spreading and non-spreading colonies of *F. columnare* and measured the expression of genes related to gliding and secretion. Colony types Rz and S responded to decreased nutrient concentration by increasing spreading behavior, but R type did not have this plastic feature, as reported previously by Laanto et al. (2012). However, mutations were not found in the studied gliding motility genes, indicating that other genes may also be needed for the formation of spreading colonies. Although a decrease in nutrients remarkably increased the spreading of colonies, it did not correspond to the expression of gliding motility genes in a uniform manner. We also found that the capacity for extracellular secretion of proteases was maintained in the non-spreading morphologies, which may denote the presence of functionally intact secretion systems.

Each colony type was viable both at low- and high-nutrient conditions, when they were cultivated in a liquid medium. Even though each colony type was more successful at a high nutrient level (in liquid), they all expressed low colony spreading when they were cultivated on agar plates (under the same nutrient conditions). This finding indicates that smaller colonies are not produced on high-nutrient -agar due to unfavorable growth conditions but rather as a result of reduced cell motility. In biofilm measurements, we found that the Rz type was the most efficient biofilm producer in the low-nutrient treatment. The S type failed to produce biofilm, although it expressed increased colony spreading under low-nutrient conditions. This indicates that spreading *per se* is not an indicator of biofilm formation. Biofilm formation is a process in which successful adhesion is required in order to attach to a surface (Garrett et al., 2008). As the biofilm formation capacity of the S type was comparable to the control (a growth medium without bacteria), it may be incapable of proper surface adhesion, possibly due to lack of functional cell surface adhesins. Indeed, the colonies of the S type are only moderately adherent (Kunttu et al., 2011) and can be removed from agar plates easily compared to the Rz and R colony types. However, the capacity for extracellular secretion, gliding, and adhesion to other cells demonstrated in the S colony type indicates that different adhesins are needed for surface adhesion and social motility of *F. columnare.* The adhesins required for *F. columnare* attachment to surfaces of different compositions (abiotic or biotic) are yet to be identified.

Microscopic microrhizoids were observed at the edge of spreading colonies. Importantly, the cells involved in microrhizoids expressed cooperative behavior in terms of social motility as they glided along neighboring cells and, thereby, mediated the spreading of the biofilm. Previously, we have proposed the involvement of social movements in *F. columnare* biofilm formation due to coordinated cell organization (Laanto et al., 2014). Indeed, bacterial pathogens are known to cooperate, especially with regards to biofilm formation (see e.g., Nadell et al., 2016). Furthermore, high nutrient levels reduced gliding behavior in biofilm. We have recently shown that a high environmental nutrient level leads to higher virulence via increased virulence factor expression (Penttinen et al., 2016). Therefore, it remains to be resolved whether motility itself is essential for *F. columnare* virulence or whether gliding motility and virulence are related solely via a common secretion route for adhesins and virulence factors.

Previous studies in *F. johnsoniae* have demonstrated that a mutation in any of the gliding motility genes results in disruption of the gliding motility machinery and the formation of non-spreading colonies (see e.g., McBride and Nakane, 2015). In order to study the genetic background of gliding motility in *F. columnare* spreading and non-spreading colony types, we sequenced genes involved in gliding motility apparatus and T9SS: *gldH*, *sprA*, *sprE*, *sprF*, *sprT*, and *porV* and genomic regions spanning *gldFG*, *gldKLMN*, and *sprCD*. Surprisingly, these genes were identical between the spreading (Rz and S) and the non-spreading (R) colony types. Furthermore, genetic differences were not found in the predicted regulatory regions. A sequence analysis of other *gld* genes could provide information on differences between the colony types, but it is possible that other genes are also involved in colony spreading. A whole genome-sequencing approach of different colony morphotypes would be efficient for identifying genetic factors that contribute to the changes in colony spreading. In *F. johnsoniae*, for example, *secDF* mutants have been shown to be incapable of gliding motility and chitin utilization and to produce non-spreading colonies (Nelson and McBride, 2006). It was hypothesized that SecDF may not be involved in gliding directly, but may have a role in translocation of GldJ (Nelson and McBride, 2006). Furthermore, transposon mutagenesis revealed that a thiol oxidoreductase-like protein, TlpB, is associated with gliding motility and virulence in *F. psychrophilum* (Alvarez et al., 2006). Thiol oxidoreductases are essential for the folding of several proteins, including those related to virulence (Fabianek et al., 2000), but their function in *F. columnare* has not been studied.

We studied the gene expression of gliding motility- or spreading-associated genes in low-nutrient (0.5x Shieh) and high-nutrient (2x Shieh) agar plates. Colony spreading increased in low-nutrient conditions and decreased in high-nutrient conditions (**Figures 1**, **2**). Expression of *gldG, gldH, gldL*, and *sprE* (in pairwise comparison, also *sprT* and *porV*, see **Tables 1**, **2**) was significantly influenced by colony type, but the highest expressions were often observed in the non-spreading R type. In general, gene expression levels were not consistently associated with the activity of gliding motility. Growth under low-nutrient conditions significantly increased expression of *sprA* in Rz and R types and the expression of *sprF* in the R type. In *F. johnsoniae*, SprA has been identified as a cell surface protein, and it has been hypothesized that it serves as a link between surface adhesins and gliding motor (Nelson et al., 2007). In that sense, increased production of SprA under low nutrient levels seems reasonable. Previously, it has been found that *F. psychrophilum* GldN expression increases *in vivo* and in iron-limited media (LaFrentz et al., 2009). Indeed, low-nutrient conditions induce motility in *F. psychrophilum* and *Vibrio parahaemolyticus* (McCarter, 1999; Perez-Pascual et al., 2009), whereas *Salmonella enterica* serovar Typhimurium and *Escherichia coli*, which express swarming motility on solid surfaces, act conversely (Harshey and Matsuyama, 1994; Toguchi et al., 2000). Combined with the observed phenotypic changes in colony spreading and gliding motility, our findings indicate that environmental nutrients may rather regulate the activity of gliding motility machinery, than the abundance of its components. While qPCR measures the quantity of a present mRNA of a target gene (Wong and Medrano, 2005), bacterial transcripts of secreted proteins may experience post-transcriptional and post-translational processing steps prior to transportation through the cell membranes. Consequently, failure in any of these processes may lead to unsuccessful protein translocation and may further alter the constitution of cell surface. Therefore, malfunctions in the T9SS may disrupt secretion and result in accumulation of secreted products in the cytoplasm or the periplasmic space (Sato et al., 2005; Shrivastava et al., 2013). It is also possible that gliding motility-related genes may be regulated via more complex pathways. In *P. gingivalis*, PorX and PorY putatively form a two-component signal transduction system that regulates the expression of a subset of T9SS genes, including *porT, sov, porK, porL, porM*, and *porN* (Sato et al., 2010), that correspond to *sprT, sprA, gldK, gldL, gldM*, and *gldN* in *F. columnare*, respectively. The gene sequences of *F. columnare porX* and *porY* orthologs were found to be identical in different colony types, which implies that the observed gene expression differences are not a result of a mutation in these genes. Hence, the role of PorX and PorY or other regulatory mechanisms that would direct gliding motility or T9SS activity remain to be studied in more detail. For example, preparing *porX* and *porY* null mutants and exploring the effect on gene expression could make an important contribution to the understanding of gliding motility and its regulation. In addition, transcriptome analysis of different strains and their colony types could provide insight into gene expression under different nutrient conditions and help to build a more comprehensive perspective on factors that are associated with colony spreading and gliding motility.

All the colony types showed proteolytic activity on high- and low-nutrient milk agar, indicating that colony spreading is not associated with secretion in this *F. columnare* strain. Previous studies have demonstrated that similarly to disruption of gliding motility, mutations in the gliding motility genes affect *F. johnsoniae* proteinase secretion (Sato et al., 2010). Therefore, our results indicate that the genes related to gliding motility and T9SS in the studied strain B067 are intact. Furthermore, no considerable differences in proteolytic activity were detected on low- and high-nutrient milk agar, though in some cases, low nutrient concentration has been shown to decrease protease activity (Newton et al., 1997). The ECP profiles of Rz, R, and S types grown in low and high nutrient conditions did not differ remarkably from each other, except for a ∼13 kDa protein band detected in ECP, which was present only in the Rz type. This protein has previously been connected to the virulent colony type (Laanto et al., 2014), but its role in pathogenicity is unknown. However, it should be noted that ECP profiles were isolated from bacteria grown in liquid media, and therefore, their ECP profiles may not fully correspond to the profiles obtained from bacteria grown on an agar plate (and which would express gliding motility more vigorously).

To conclude, environmental nutrients are important regulators of *F. columnare* gliding motility and the expression of associated genes. Despite the mounting data on *Flavobacterium* gliding motility and T9SS, the knowledge of their regulatory pathways is limited, and how environmental cues contribute to the regulation of these signaling pathways in *F. columnare* remains to be determined. Therefore, the possibility cannot be ruled out that differentially spreading morphotypes of *F. columnare* are caused by factors that are yet to be identified. Understanding the differences between spreading and non-spreading morphotypes may help to disentangle factors related to gliding motility and virulence in *F. columnare*.

## Material and Methods

### Bacterial Strains and Growth Conditions

In all experiments in this study, we used three different colony types (rhizoid [Rz], rough [R] and soft [S]) of *F. columnare* strain B067 (Laanto et al., 2011). The bacterial strain was originally isolated from a trout that was killed during a columnaris disease outbreak at Finnish fish farm (Laanto et al., 2011). The R colony type was obtained after exposing the original Rz isolate to bacteriophages (Laanto et al., 2012). The S type appeared spontaneously during laboratory culture of the Rz type (Laanto et al., 2014).

Bacterial stocks were stored at -80°C in 10% fetal calf serum and 10% glycerol and revived from the freezer in fresh modified Shieh medium according to Song et al. (1988), which is referred to as Shieh medium in this study and is used as a base of nutrient-modified media. In nutrient-modified media (0.5x or 2x Shieh), all the ingredients were either halved or doubled. In 0.5xN and 2xN Shieh media, only the concentration of peptone and yeast extract were halved or doubled, respectively (for detailed compositions of the media, see Penttinen et al., 2016). After revival from the freezer, liquid bacterial cultures were cultivated at RT/26°C, with agitation of 115/150 rpm for 24–48 h to obtain dense cultures. Cultures were refreshed with fresh 1x Shieh medium, and cultivation was continued for 16–24 h. For plate cultures, dense liquid culture was streaked on a Shieh agar plate, which was incubated at RT for 2 days.

### Colony Morphology, Growth, and Biofilm Formation in Different Nutrient Conditions

Liquid bacterial cultures of Rz, R, and S grown in 1x Shieh were streaked on 0.5xN, 1x, and 2xN Shieh plates. Plates were incubated for 2 days at RT, after which the colony morphology was determined.

In order to evaluate the bacterial viability in different nutrient concentrations, 1.18 × 10^7^–1.28 × 10^7^ colony forming units of B067 Rz, R, and S in a total volume of 400 μl containing 0.5xN, 1x, or 2xN Shieh (*N* = 8) was cultivated on Honeycomb 2^®^ microplate (Growth Curves Ltd.) in a Bioscreen C^TM^ spectrophotometer (Growth Curves Ltd.) at 26°C. The absorbance (600 nm) was measured every 5 min for 65 h. The viability was estimated as the maximum absorbance recorded during the cultivation.

The biofilm formation capacity under various nutrient conditions was determined by cultivating 1.7 × 10^6^-1.83 × 10^6^ colony forming units of B067 Rz, R, and S in a total volume of 100 μl in 0.5xN and 2xN Shieh media on a Maxisorp plate. After a 44-h -incubation at RT, the emptied wells were rinsed twice with 200 μl of phosphate buffer solution (PBS). The biofilm-forming bacteria were stained with 125 μl of 0.1% crystal violet solution for 10 min and rinsed three times with 200 μl of PBS and the plate was dried at RT overnight. To solubilize the crystal violet, 125 μl of 96% ethanol was added. Finally, 100 μl of the solution was transferred to a fresh microplate, and absorbance was determined at the wavelength of 595 nm with a Multiskan FC spectrophotometer (Thermo Scientific).

### Imaging Bacterial Cell Movements

*Flavobacterium columnare* B067 Rz cells were scratched from 0.5xN and 2xN Shieh agar plates and suspended in 0.5xN and 2xN Shieh liquid media, respectively. The cell suspension was pipetted and imaged on an eight-chambered ibidi^®^ ibiTreat μ-Slide (ibidi GmbH) covered with CID lid for μ-dishes (ibidi GmbH). The bacterial cells were imaged with a Nikon AR1 laser scanning confocal microscope using a 488 nm Argon laser and CFI Apo VC 60x water immersion objective (numerical aperture 1.2).

In order to image the spreading behavior of the different colony types, 3–5 μl of overnight culture of *F. columnare* B067 Rz, R, or S was pipetted onto an eight-chambered ibidi^®^ ibiTreat μ-Slide (ibidi GmbH) between the bottom of the chamber and a 0.5xN, 1x, or 2xN Shieh agar layer. The bacteria were cultivated overnight at RT, after which the motility on the edge of the spreading colony was imaged as described above. In order to make slow bacterial movements detectable to the human eye, the videos were sped up as follows: Supplementary Videos 1–8: 4×; Supplementary Video 9: 1,800×.

### Preparation of the Samples for Gene Expression Analysis

Several dilutions (with Shieh media) were made from liquid bacterial cultures, which were then spread on 0.5x and 2x Shieh agar plates in order to obtain plates with separate colonies and on which the colony types were recognizable. Plate cultures were incubated at RT for 2 days, after which each plate was inspected to ensure it contained only the appropriate colony type. By diluting the bacterial cultures, close to round-shaped colonies were observed and their size was measured. A colony was considered to be the area covered with bacterial cells, including both the denser area in the middle of the colony (if present) and the more transparent area around it. Following the manufacturer’s instructions, the bacterial colonies were suspended in RNA Protect^TM^ Bacteria Reagent (QIAGEN), which protects RNA from degradation. Total RNA was extracted from Rz, R, and S colonies grown on 0.5x and 2x Shieh agar plate cultures with an RNeasy^®^ Mini Kit (QIAGEN). If there was any remaining genomic DNA, DNAse treatment with DNA-free^TM^ (Ambion by Life Technologies) was carried out. RNA quality was verified by running the samples on an Agilent RNA 6000 Nano Chip (Agilent Technologies) in an Agilent 2100 Bioanalyzer (Agilent Technologies) and determining the RNA integrity number (RIN) for each sample. Only qualified samples (RIN above 8.7) proceeded to cDNA synthesis which was performed immediately after RNA validation. RNA was reverse-transcribed into cDNA in triplicate reactions with iScript^TM^ cDNA Synthesis Kit (Bio-Rad) according to the manufacturer’s instructions. cDNA reactions with a volume of 20 μl contained 1x iScript^TM^ reaction mix, 1 μl iScript^TM^ reverse transcriptase, and 40 ng of template RNA. Replicate reactions were pooled and used as a template in qPCR.

### RT-qPCR

Each 20 μl qPCR reaction, run in triplicates, contained 40 ng (*gapdh, glyA, gldG, gldH, gldL* and, *sprB*) or 80 ng (*sprA, sprE, sprF, sprT* and *porV*) of cDNA template, 0.5 μM of both forward and reverse primers and 1X iQ^TM^ SYBR Green Supermix (Bio-Rad) that contained iTaq DNA polymerase (25 U/ml). qPCR reactions conditions were as follows: 95°C for 3 min, followed by 40 cycles of 95°C for 10 s, Tm °C for 20 s and 72°C for 20 s, [melting temperature (Tm) was chosen according to the primer pair (**Table 3**)]. CFX96^TM^ Real-Time System C1000^TM^ and C1000^TM^ Touch Thermal Cyclers (Bio-Rad) were used in qPCR plate runs on 96-well Hard-Shell^®^ PCR plates (Bio-Rad). On each plate, two interplate calibrator samples in triplicates were run to normalize interplate variation.

**Table 3:** Primer sequences and properties used in RT-qPCR study of *F. columnare*.

Primer	Sequence	Amplicon Length	Tm (°C)	Efficiency (%)	Reference
FC_gap1_fwd	ACCATCCCAAACAGGAGCCGC	98	56	105.7	Penttinen et al., 2016
FC_gap1_rev	CGTCTGCTGTAGGTACGCGCA				Penttinen et al., 2016
FC_glyA_fwd	CCAAACCCTTGGGGCTATACAACCC	98	60	102.8	Penttinen et al., 2016
FC_glyA_rev	AGAGGGCCTCCTTGATTACCTGGAA				Penttinen et al., 2016
FC_gldG_fwd	AGCAGAAGCAGTGATGCAGCA	125	58	100.95	This study
FC_gldG_rev	TGCCTTTGTAGGTAGCAATAGCCCA				This study
FC_gldH_fwd	CTTTGAAAACGGATGGCC	221	56	99.15	Klesius et al., 2010
FC_gldH_rev	CTTGCCCCATAAGACTTCC				Klesius et al., 2010
FC_gldL_fwd	GCAAGCGCTATGCTTATTGCTGGT	131	58	101.4	This study
FC_gldL_rev	GCAGTTGGTTGTCCCCCTGCT				This study
FC_sprA_fwd	GCAGAAAATGTTTGGCCCGT	162	60	99.95	This study
FC_sprA_rev	ACCGGCAGTTGCTCCATTAT				This study
FC_sprB_fwd	ACCAGCTGCTCCATGGTCAACTAC	157	60	100.1	This study
FC_sprB_rev	CGAAGGTGTCGTAGGGGCCG				This study
FC_sprE_fwd	AGCCGTGCAGAAGATAAAGC	151	60	100.8	This study
FC_sprE_rev	ACGCTTCTAATGCGGGTACAA				This study
FC_sprF_fwd	AGTCGTCAAATGGGGGCTAA	148	60	99.65	This study
FC_sprF_rev	TCACGCTTCCATCAAAGGTT				This study
FC_sprT_fwd	AACCAGGACTGCATTACGGA	144	60	101.1	This study
FC_sprT_rev	GCTTGATGTTACCTGTGCGTT				This study
FC_porV_fwd	GTGCCAACTCCTAAAACAGCC	152	60	96.85	This study
FC_porV_rev	AAACCTCCTGGAGCATCACC				This study


Primer-BLAST^1^ was used to design a primer pair for each target gene. Primers used in this study are presented in **Table 3**. The specific binding of each primer pair was tested by checking the amplicon length on agarose gel and with melt curve produced by CFX Manager^TM^ Software v3.0. *glyA* and *gapdh* have been qualified as valid and stably expressed in each *F. columnare* colony type in various nutrient conditions (Penttinen et al., 2016). They were, thus, used as reference genes to normalize the gene expressions of gliding motility-associated genes. *M*-value (Vandesompele et al., 2002), which indicates gene expression stability, was measured for the current dataset for reference genes *glyA* and *gapdh* using CFX Manager version 3.0 (Bio-Rad).

### Relative Quantities

For the following data prehandling, GenEx version 6.0 (MultiD Analyses) was utilized. Any missing quantification cycle (Cq) value was replaced with the average Cq of its two qPCR replicates. IPC samples run on each plate were used to minimize variation between different plate runs. Efficiency for each primer pair was calculated from a standard curve (with CFX Manager version 3.0) and Cq values were corrected with the efficiency within each gene. The averaged Cq values were normalized with reference genes and transformed into relative gene expression with GenEx version 6.0 (MultiD Analyses).

### Statistical Analyses

The effect of colony type and nutrient level on gliding motility gene expression was tested with ANOVA. *Post hoc* tests were Bonferroni-corrected. Data for *sprA, sprE*, and *gldG* were log-transformed to fulfill the assumption of normality and homoscedasticity. Statistical analyses were performed with IBM^®^ SPSS^®^ Statistics 22 (IBM Corporation), except for *gldL*, which could not be transformed to fulfill the assumption of ANOVA and was, thus, analyzed by ARTtool package in R (version 3.1.3) (Wobbrock et al., 2011). Pairwise comparisons were not performed for *gldL*.

### DNA Sequencing of Gliding Motility Genes in Different Colony Types

Genomic DNA of *F. columnare* strain B067 colony types Rz, R and S was extracted from bacterial liquid cultures grown overnight in 1x Shieh medium at RT (115 rpm) using a GeneJET Genomic DNA Purification Kit (Thermo Scientific). The genes related to gliding motility, T9SS or their regulation (*gldH*, *sprA*, *sprE*, *sprT*, and *porV)*, genomic regions spanning *sprCDBF, gldFG* and *gldKLMN* as well as partners of a putative two-component system, *porX* and *porY*, were first amplified with PCR using genomic DNA of B067 type Rz, R or S as a template. The 20 μl reactions were performed using Phusion Flash High-Fidelity PCR Master Mix (Thermo Fisher Scientific), with primer concentrations of 0.5 μM and a template amount of 1–10 ng per reaction. The PCR protocol described by the manufacturer was followed, taking into account the differences in primer melting temperatures and PCR product sizes.

The organization of the genes studied in RT-qPCR assay within operons was predicted with DOOR2 (Dam et al., 2007; Mao et al., 2009) according to the genome sequence of *F. columnare* ATCC 49512. The genomic region upstream of the predicted operon was assumed to contain the appropriate promoter region. The upstream regions of operons *gldFG*, *gldKLMN*, and *sprCDBF*, those operons comprising genes *gldH*, *sprA*, or *porV* as well as the upstream region of the gene *sprE* (which was predicted to be expressed alone) were sequenced in B067 Rz, R, and S types.

Prior to sequencing the PCR products were purified using QIAGEN’s QIAquick PCR Purification Kit. Primers for the sequencing reactions were designed in 500 bp intervals using VectorNTI version 11.5.1 (Invitrogen), utilizing our shotgun sequencing results as a template. A BigDye Terminator v3.1 Cycle Sequencing Kit (Applied Biosystems) was used for sequencing the DNA fragments using the Sanger sequencing technique with an automated sequencing instrument 3130*xl* Genetic Analyzer (Applied Biosystems). The identity of each base was determined with at least two good quality reads. Basecalling was done using Sequence Analysis 6 (Applied Biosystems). Gene sequence assembly and the alignment of homologous sequences of different colony types were performed with Geneious 8.1.5 (Biomatters Ltd.). The assembled gene and regulative region sequences of Rz, R, and S colony types are found in GenBank (accession number in brackets): *gldFG* (MF278296), the upstream region of operon comprising *gldH* (MF278297), *gldH* (MF278298), *gldKLMN* (MF278299), the upstream region of operon comprising *sprA* (MF278305), *sprA* (MF278306), *sprCD* (MF278307), *sprE* (MF278308), *sprF* (MF278309), *sprT* (MF278300), the upstream region of operon comprising *porV* (MF278301), *porV* (MF278302), *porX* (MF278303), and *porY* (MF278304).

### Protease Activity and ECP Production in Different Nutrient Concentrations

To study the effect of nutrient level on proteolytic activity, B067 colony types Rz, R, and S were cultivated in 0.5xN and 2xN Shieh media and 10 μl of bacterial culture (containing 1.4 × 10^6^ CFUs ±4 × 10^4^ SE on average) were spotted, respectively, on 0.5xN or 2xN Shieh agar plates containing 1.5% skim milk (Merck). Plates were incubated for 2 days at RT, after which the clear zone (indicating proteinase production) around the bacterial growth was detected.

Extracellularly secreted product samples were prepared as follows: 8 ml from *F. columnare* Rz, R, and S liquid cultures were added to 100 ml of fresh 0.5xN and 2xN Shieh media. The cultures were grown for 19 h. One hundred milliliters of dense bacterial culture was centrifuged at 4°C (4,500 rpm, 15 min). The supernatant was first filtered through a 0.45 μm Supor^®^ membrane (Pall Corporation) and then concentrated with 10 K Amicon Ultra-15 Centrifugal Filter Units (Merck Millipore) at 4°C to final volume of 2–3 ml. ECP samples were divided into 500 μl aliquots and stored at -20°C. Protein concentration of the ECP samples was determined using the Bradford method (Bradford, 1976) against a standard curve made with known amounts of bovine serum albumin. Fifty micrograms of each ECP sample (except 150 μg of Rz grown in 2xN Shieh) was loaded onto 14% Tricine-SDS-PAGE gel. The gel was run for 24 h at 90 V/30 mA and stained with Coomassie Brilliant Blue solution.

## Author Contributions

RP and L-RS designed the study. RP and VH conducted the laboratory experiments. RP, L-RS, and VH wrote the manuscript.

## Conflict of Interest Statement

The authors declare that the research was conducted in the absence of any commercial or financial relationships that could be construed as a potential conflict of interest. The reviewer JW and handling Editor declared their shared affiliation.

## Footnotes

1 http://www.ncbi.nlm.nih.gov/tools/primer-blast/
