# Supplementary material for: Gliding Motility and Expression of Motility-Related Genes in Spreading and Non-spreading Colonies of Flavobacterium columnare
Source: Front Microbiol. 2018 Mar 26;9:525. doi: 10.3389/fmicb.2018.00525 (PMC5879114; doi:10.3389/fmicb.2018.00525)
Supplement: Supplementary file 10 [file Data_Sheet_1.docx]

Supplementary Material

Gliding motility and expression of motility-related genes in spreading and non-spreading colonies of *Flavobacterium columnare*

Reetta Penttinen*, Ville Hoikkala and Lotta-Riina Sundberg

*** Correspondence:** Reetta Penttinen: [reetta.k.penttinen@jyu.fi](mailto:reetta.k.penttinen@jyu.fi)


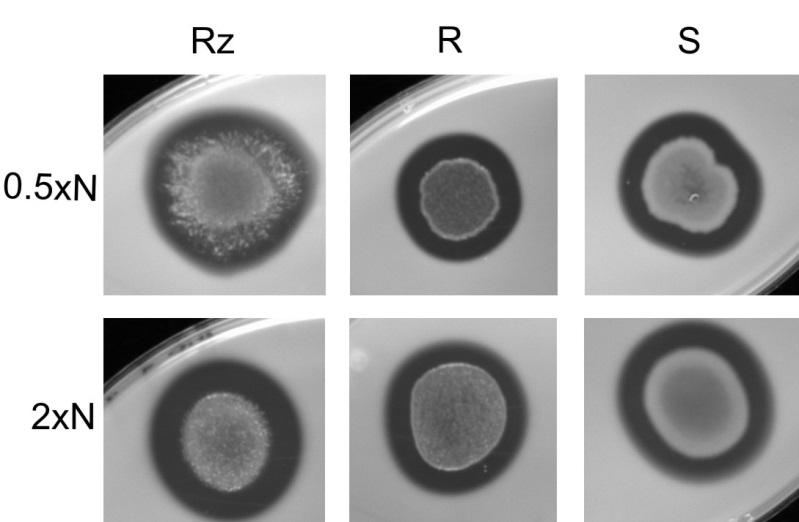


**Supplementary Figure S1.** Proteolytic activity of *F. columnare* B067 colony types Rz, R and S grown on 0.5xN and 2xN Shieh milk (1.5 %) agar plates. The peptone and yeast extract concentrations were either halved (0.5xN) or doubled (2xN) compared to normal Shieh medium.

**
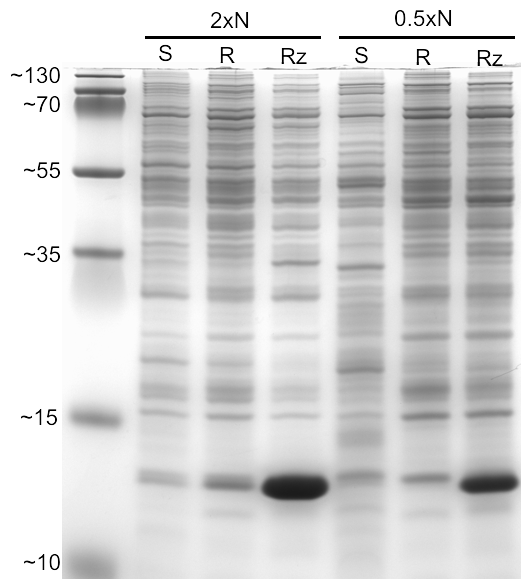
**

**Supplementary Figure S2.** Extracellularly secreted products of *F. columnare* B067 colony types Rz, R and S grown in liquid 0.5xN and 2xN Shieh media.

**SUPPLEMENTARY VIDEOS**

**Supplementary Video 1.** Motility of *F. columnare* B067 Rz cells grown on 0.5xN Shieh agar. Scale bar 10 µm.

**Supplementary Video 2.** Motility of *F. columnare* B067 Rz cells grown on 2xN Shieh agar. Scale bar 10 µm.

**Supplementary Video 3.** Colony spreading of *F. columnare* B067 Rz grown on 0.5xN Shieh agar. Scale bar 10 µm.

**Supplementary Video 4.** Colony spreading of *F. columnare* B067 Rz grown on 2xN Shieh agar. Scale bar 10 µm.

**Supplementary Video 5.** Colony spreading of *F. columnare* B067 R grown on 0.5xN Shieh agar. Scale bar 10 µm.

**Supplementary Video 6.** Colony spreading of *F. columnare* B067 R grown on 2xN Shieh agar. Scale bar 10 µm.

**Supplementary Video 7.** Colony spreading of *F. columnare* B067 S grown on 0.5xN Shieh agar. Scale bar 10 µm.

**Supplementary Video 8.** Colony spreading of *F. columnare* B067 S grown on 2xN Shieh agar. Scale bar 10 µm.

**Supplementary Video 9.** Colony spreading of *F. columnare* B067 Rz growing 8 hours on 1x Shieh agar. Scale bar 20 µm.
